# “You Sort of Go Down a Rabbit Hole...You’re Just Going to Keep on Searching”: A Qualitative Study of Searching Online for Pregnancy-Related Information During Pregnancy

**DOI:** 10.2196/jmir.6302

**Published:** 2017-06-02

**Authors:** Julie Prescott, Lynn Mackie

**Affiliations:** ^1^ Education and Psychology University of Bolton Bolton United Kingdom

**Keywords:** pregnancy, information seeking behavior, qualitative research

## Abstract

**Background:**

The Web is becoming increasingly popular for gaining information on medical or health issues; with women in particular likely to search online for this type of information and support. Despite the increased use of the Web for health-related information, we need to question whether the Web and the ease of seeking health information that it provides leads to more (patient) empowerment. As well as being a time of joy and expectations, pregnancy can be a worrying time for women, especially first time mums-to-be, with unfamiliar experiences and symptoms and concerns for the baby as well as the self.

**Objective:**

Our aim was to explore how and why pregnant women use the Web to gain information and support during pregnancy and what they consider a reliable source.

**Methods:**

To meet the objectives of the study, a qualitative approach was required to gather information on the experiences of currently pregnant women who use the Web to gain information and support during their pregnancy. Sixteen pregnant women took part in a semistructured interview, either face-to-face or via telephone. The interviews took place from January to March 2016, all participants were from England, and the health professionals are all employed by the National Health Service (NHS). Qualitative analytical procedures were employed using inductive thematic analysis supported by NVivo software (QSR International).

**Results:**

Pregnant women found reassurance from the experiences of others. This reassurance resulted in them feeling less alone, as well as enabling them to normalize any symptoms or experiences they were undergoing. The women understood that caution was needed at times while reading the stories of others, acknowledging the potential for extreme cases or worst case scenarios. This is particularly pertinent to the Web, as this wide range of stories may not be as easily accessible if stories where confined to those in a woman’s offline social circle. The interviews provide insights into how and why pregnant women search online for information and perhaps more so, support while pregnant.

**Conclusions:**

Searching for health information and advice online during pregnancy is viewed as quick, easy, and accessible. The affordances of the Web have provided women the opportunity to go online as a first port of call. Knowing they were not alone and reading the experiences or symptoms of other pregnant women enabled women to normalize their experience and was ultimately reassuring for pregnant women.

## Introduction

The Web is becoming increasingly popular for gaining information on medical or health issues [1]. Indeed, according to a Pew Internet report, 80% of American Web users look for health information online [2], and 35% of American adults have gone online to diagnose a medical condition, referred to as “online diagnosers” [1]. Women are more likely to go online to figure out a possible medical diagnosis than men [1] and search online for health information [3]. However, despite the increased use of the Web for health-related information, we need to question whether the Web and the ease of seeking health information that it provides leads to more (patient) empowerment. Women are more likely to search for health information online [3] for themselves and other family members, and as previously mentioned they are also more likely to go online to try and diagnose a medical condition [1]. A large Australian study [4] found that women who experienced stigmatized conditions such as sexually transmitted infections or mental health were more likely to search online for health information [4]. Women also use the Web to seek information regarding pregnancy and childbirth in particular, as these are often worrying times for women [3,5]. Research suggests that health anxiety is not elevated during pregnancy, although health anxiety is higher for those women who have experienced complications during pregnancy [6].

As women utilize the Web for health information more so than men, and pregnancy and childbirth can be worrying times for women, it was deemed important to consider how women use the Web during pregnancy for pregnancy-related information and how they negotiate and navigate through the mass of data available to them online. The vast amount of information and personal stories available and easily accessible online suggest that women must negotiate what information they take on board and how they react to the “horror stories” due to numerous risks involved in pregnancy and childbirth [7].

Previous research has found that around 80% of Web users seek health information online [8,2] for reasons such as convenience, anonymity, specialized advice, and social support when feeling stressed or worried [9]. Previous research has focused on parents who seek health information online for their children [10]. Bernhardt and Felter [10] found that women as parents are among the highest information-seekers who use the Web to confirm beliefs or get a “second opinion.” They also found that online sources need to be clear with advice in order to gain more trust from mothers, and more trusted advice comes from clinical professionals or parents (in online blogs). The parents in the study briefly discussed their pregnancy health information seeking behavior, stating that they used the Web for support and health advice [10].

In a more recent study, Lagan et al [5] aimed to understand how and why pregnant women used the Web for health information and how this impacted on their decision making during pregnancy. The findings revealed that women googled information and used the information found online to supplement offline information they had received from health professionals, and the Web played a significant part in health information-seeking and decision-making during pregnancy. From the findings, the authors were left with questions with regard to the knowledge of women to evaluate the quality of the information sourced. In a 2011 study [11], Lagan et al investigated the perspectives of midwives toward the online searching behavior of pregnant women, finding a general increase in the use of the Web among pregnant women, with midwives concerned about the accuracy of information found, corroborating the authors earlier findings.

It is viewed as important that people know where to seek information online; having this knowledge could empower people and health care providers should suggest suitable sites for pregnancy-related information [12]. This is a view shared by Lagan et al [5]. In their study of online pregnancy-related information for nutrition and physical exercise, Huberty et al [12] found that the information women sourced via the Web during their pregnancy increased their confidence in decision-making during pregnancy. However, it was acknowledged that there is a need to question the trustworthiness of information found online compared to offline sources such as doctors or family and friends. Research has found that information sought and found online is secondary in both priority and reliability toward offline sources [5]. People in the digital age want to be informed patients and online health information searching is not due to any dissatisfaction with doctor’s care (or any other health care professional [HCP]) or information provided [5,13].

Supporting this view, Song et al [7] found that although women use the Web, they still rely on their doctors. Women utilized the Web to confirm normalcy and help them take control. They used the online information to confirm knowledge received from health professionals and to seek reassurance of what is normal, with a general desire not to be the only one experiencing symptoms; this was found to be especially pertinent for first time mothers. The study found that pregnant women were interested in keeping track of fetal development [7], supporting early research that fetal development is the most cited online search topic of interest for pregnant women [10].

The overall aim of the larger study was to gain an understanding of how pregnant women use the Web for health-related information during pregnancy, as well as to gain an understanding of how women can skillfully surf in order to reduce any potential anxiety caused through seeking health information online. This paper focuses on how and why pregnant women use the Web to gain information and support during pregnancy and what they consider a reliable source.

## Methods

The larger study from which this paper is drawn took a mixed-methods approach, using both qualitative semistructured interviews with pregnant women and a Web-based questionnaire for currently pregnant women and new parents, both male and female, in order to understand their online health information-seeking behavior. The interviews and questionnaire elements of the study ran simultaneously in order for a larger, more diverse dataset from which to derive results. This paper focuses on the findings of the qualitative element of the study.

### Sampling and Participants

Using a snow-ball sampling technique, a total of 16 semistructured interviews were conducted with pregnant women. n order to recruit pregnant women to the study, the study was advertised around the university, and participants were also asking for referrals of other currently pregnant women. Although small, the sample size was deemed adequate due to data saturation [14]. Data saturation was reached when there was enough information to replicate the study [15,16]; new information was not obtained [17] and further coding was not feasible [17], and future interviews would not yield further information or coding categories [17].

All interviews were conducted by the same interviewer. The interviewer was trained in qualitative research. The interviews ranged in duration from 20-60 min and were conducted one-to-one either by phone or in person from January to March 2016. Of the 16 women interviewed, only 14 provided demographic data (see Table 1 for information on participants). Participants received and were asked to read a participant information sheet informing them about the study and what was required of them. All participants were required to sign a consent form before the interviews took place. The consent form informed participants that the interview was voluntary and that they were free to stop the interview at any time during the interview, without giving a reason as to why. All participants were also required to complete a demographics form asking information about age and ethnicity. The form also sought information related to the women’s current pregnancy, such as trimester, any pregnancy related and nonpregnancy related medical conditions, and use of the Web and support networks.

The interview schedule consisted of 4 sections. Section 1: online information seeking, with 7 questions (ie, what online sources do you use to find out information about your pregnancy?). Section 2: how you search, with 5 questions (ie, how do you know what to trust online?). Section 3: impact of online searching, with 9 questions (ie, what makes online information reassuring? How long does that last? What makes online information worrying? How long does that last?). Section 4: support and coping, 6 questions (ie, do you ever access any online health care professionals?).

### Ethical and Research Approvals

Approval for the study was given by University of Bolton Research Ethics Committee in October 2015, United Kingdom. 

### Data Analysis

All interviews were digitally recorded and transcribed verbatim. All qualitative data were analyzed using Nvivo version 10 software. The qualitative approach used to analyze the interviews was an inductive thematic analysis [18, 19] since the analysis was grounded in the data rather than existing theories. The data was analyzed using Braun and Clarkes approach [18] for using thematic analysis in psychology. In order to ascertain and increase intercoder reliability and the reliability of the results, 2 transcripts where independently coded by both authors to develop a coding framework and code book. Any subsequent additional theming was discussed during independent analysis. The stories from other women who had experienced the same or similar experiences helped pregnant women to feel reassured and less like they were the only ones going through something.

## Results

### Overview of Results

In general, pregnant women tended to find the Web a useful source of information and advice during their pregnancy. Although there were things that worried them when they searched on the Web, overall the accessibility of Web-based information and the stories and experiences of other women were viewed as reassuring. Four main themes emerged from the interviews. Table 2 shows the main themes, topics, and an example quote from the interview analysis.

### Online Forums: Reading the Stories and Experiences of Other Pregnant Women

From the analysis, it was evident that the participants tended to search and engage with online forums to read and get advice from other pregnant women. All the women talked about other women’s stories they read on the Web and these were viewed positively and seen as providing pregnant women with reassurance.

#### Reassurance, Not Alone, and Normalized

Other women’s stories and experiences had a positive impact in terms of reassurance and helping to reduce worry as shown in Table 2. The stories from other women who had experienced the same or similar experiences helped pregnant women to feel reassured and less like they were the only ones going through something alone. Not feeling as if they were the only one’s experiencing something also normalized symptoms and issues for women as highlighted in the quotes in Table 2. Interestingly, the following quote shows how some women found reading other women’s experiences as supportive and which enabled them to ask questions they did not want to ask their midwife:

If I had to ask the midwife like everything I was worried about, I wouldn’t worry her that much and ask her that. And I think also the fact that there’s so many people who are talking about their own experience, so it can be really supportive so it’s like if you’re worried about your next scan or whatever it is then you can do a post saying I’m worried has anyone else been, and then you’ve got lots of people who kind of supporting you.

Participant 14, multiparous

**Table 1:** Demographics or characteristics of participants.

Demographics		n
**Age in years**		
	16-20	1
	21-25	6
	26-36	2
	31-35	3
	>36	2
**Pregnancy status**		
	Primiparous	6
	Multiparous	8
**Trimester at the time of interview**		
	1	0
	2	7
	3	7
**Medical complications in this pregnancy (including** **pre-eclampsia, depression, diabetes, and sickness)**		
	Yes	7
	No	7
**Medical complications in a previous pregnancy**				
	Yes	4
	No	10
**Web use**		
	Daily	12
	A few times a week	2
**Support during pregnancy**		
	Midwife	14
	General practitioner	7
	Family	14
	Friends	12

Forums were also viewed by women as a place to gain information and keep up to date:

Like you know again if you think they might be a bit of new information out or, another person might have written online that they’ve got the same illness that day and you know you want to just read up about them and what they’re going through and how they’re feeling so.

Participant 1, primiparous

Others viewed searching online as a specific place for seeking reassurance rather than information as expressed in the following quote:

I always go on for reassurance really rather than getting the information. That’s what I use it for.

Participant 9, multiparous

**Table 2:** Themes and topics.

Theme	Topics	Example quote
**Sharing experiences**	Reassurance	*I’d say they reassure me, because you know I’ll search for if I’m feeling down and you’ll see other women that feel the same as you.* [Participant 1, primiparous]
	Not alone	*Just knowing somebody else has been through the same kind of thing as you and you’re not on your own, knowing that you’re not the only person going through a certain thing.* [Participant 4, primiparous]
	Normalize	*I mean there’s a lot of stuff on there, on Netmums...and when I went on Netmums there is a lot of women out there who say it’s you know—* *perfectly normal, not to worry about it. And then I did check it with my midwife and it is you know just part of the babies growing development.* [Participant 16]
	Reliability of information	*I tend to not rely on the information too much because there are you know anybody can put anything on the internet and it’s all sorts of blogs and it’s err, so yeah I do like to read opinions of other people but I don’t, I try not to depend on them.* [Participant 5, primiparous]
	Worst case scenarios	*The fact that say for example on the forum you’re not going to get people going on saying I’m having a perfectly healthy pregnancy, you’re going to have the people that have had problems. So the fact it’s almost going to kind of over-represent, it’s always going to over-represent the people who’ve had issues.* [Participant 14, multiiparous]
**Affordance of the Web**	Quick and accessible	*Well it’s immediate so you’re not concerned until you receive or get a doctor’s appointment, so you can pretty much get an answer straight away can’t you, I think it’s a really useful source* [Participant 2, multiparous]
	Not a substitute for midwife	*Online used for (short term) reassurance but offline for definitive answers, so I would trust kind of forums like Mumsnet and Netmums and things if it was just about how people felt about something. But if I actually wanted an answer to something I wouldn’t trust them. I’d trust it if it was a website where, someone would like BabyCenter I think it’s called, where someone’s asked a question and they’ve had approved people answering it. Whereas if I’m just kind of looking for reassurance like is there anyone else who was on anti-depressants and their baby was fine, then I’m more reassured by kind of anecdotal accounts for that.* [Participant 14, multiiparous]
	Good source of information and advice	*I generally actually tend to go online and like find out information first for myself before I go, go and speak about it with other people. I don’t, because that’s, that’s sort of like my nature that I feel like I bother people if I ask them something so I try not to ask questions I try to find things for myself.* [Participant 5, primiparous]
**Pregnancy-specific focus**	Search on the Web now pregnant	*I’ve looked more and researched more about pregnancy since I’ve been pregnant but beforehand I wouldn’t say I used the website if I was poorly, if I was poorly I just used to go to the doctors. I wouldn’t try and erm, you know, think of what condition I’ve got, I’d just go straight to the doctors.* [Participant 8]
	Baby development	*Online like I said I just use my app, I sometimes use google but it’s more just the app to be honest with you, just having a nosey at the changes to the baby and things like that.* [Participant 4, multiparous]
	Reliable sources	*I usually go refer back to the NHS pages as well.* [Participant 9, multiparous]
**When to stop**	Information not helping	*Just when you feel like you now well this is not really helping me anymore so just shut it down.* [Participant 5, primiparous]
	Saturation	*I think the more that the same thing that comes up then I trust it, so the more it occurs.* [Participant 9, multiparous]
	Reassured	*I just think when you feel like you’ve got an answer.* [Participant 2, multiparous]

#### Questions of Reliability

Despite the benefits gained from reading other women’s pregnancy stories and experiences, there was the recognition by the participants that there were issues with searching and reading online and caution should be taken as expressed in the following quote on the unreliability of information sourced online. This caution is due to the Web s’ often contradictory nature, and the fact that information is based on peoples, often limited experiences and are opinion based, rather than always fact based.

Contradicts itself a lot, some people say “oh I was bleeding, I didn’t have a miscarriage,” “I was bleeding, I did have a miscarriage.” So you never know what to believe. So then if a load of people have said “oh I’ve had a miscarriage these pains the same as you, I had a miscarriage,” you’re going to think you’re going to have one even though you might not. So that can really worry some people.

Participant 10, primiparous

#### Worst Case Scenarios

There is a suggestion that stories on the Web are biased toward the negative view point and there was a strong acknowledgment throughout the interviews that many of the stories people posted online were often “horror stories” or worst case scenarios and viewed as something one would not necessarily have access to if it was not for the Web. This perhaps highlights that although easily accessible, the Web is not constrained by geographic boundaries.

Because you can see obviously worst case scenario and people that might then think that it’s worse than it actually is. You know, and it isn’t always reliable is it, so and I’m not a chatroom person at all but sometimes you just referred to these aren’t you because that specific symptom you mentioned. And I think the people that generally write on these chatrooms are the people that have had the bad thing happen to them, you know the worst case scenario. You’re rarely going to hear off somebody that actually it turned out fine.

Participant 2, multiparous

These worse case scenarios or extreme cases would often cause concern for participants and increase worry in pregnant women as acknowledged clearly by participant 1:

I suppose when you read things that you know aren’t particularly nice, you know again with my pre-eclampsia you know and then if I read, you know a lady passed away from it or you know someone’s had life-long effects from it and stuff like that then you obviously, then I’m going to worry...and think you know well that could happen to me or you know well what if it does when really the chances of that are quite slim, but I suppose it is just the things you read and the things that has happened to people.

Participant 1, primiparous

### Online Affordances

#### Quick and Accessible

The affordances of the Web were viewed as important especially in terms of the accessibility and the speed with which one can get answers to concerns or worries online. This was especially important when it may be difficult to access offline health professionals. A number of the pregnant women in this study stated that they accessed the Web daily for nonwork issues, suggesting they are regular Web users. Supporting the literature that the Web is becoming increasingly popular for health information [1], participant 15 expresses this point: “When you’re looking for something you just search online.” The women tended to google symptoms as their method of searching symptoms, as research has also previously found [7], with the same participant stating: “If I’ve got pains or anything like that I’ll just Google it. Yeah so Google’s a brilliant thing.”

#### Online Information Not Viewed as a Substitute to Offline Health Professional (Midwife)

In terms of information online, participants recognized that the information received online should not be trusted and that you needed to also go offline. Searching for information online was not a substitute for seeking offline support or advice, more an accessible, easy first port of call as highlighted by the quote in Table 2.

#### Good Source of Information and Advice

Despite the acknowledgment of caution required when seeking information online, the Web was viewed as a good place to get advice and perhaps gain a feeling or sense of control with their pregnancy. In particular, it was viewed by participants as a good place to get information before seeking help from a health professional, again linking into the accessibility of online information.

In many cases it was also down to the fact that they did not want to bother health professionals, and women felt comfortable gaining reassurance or being more informed before they contacted a health professional such as a midwife.

Yeah, well, err I think, I generally actually tend to go online and like find out information first for myself before I go, go and speak about it with other people. I don’t, because that’s, that’s sort of like my nature that I feel like I bother people if I ask them something so I try not to ask questions I try to find things for myself.

Participant 5, primiparous

Interestingly, this participant felt the information sought online encouraged her to seek help offline:

Yeah, erm like the preeclampsia one last week. That scared me. Just because it’s, I’m only 28 weeks now, I was 27 last week. And to have a baby at 27 weeks I was like I’m not ready, like there’s no way this baby is ready to come out so I really hope it’s not that. Erm, so that probably worried me, so like the night before, and then I was at the doctors the next day. But I think if I hadn’t of read that I wouldn’t have been at the doctors the next day. I wouldn’t have been so worried, if that makes sense? Like I would have been ignorant in a way.) The fact that if like it is scary, so like with the, I hadn’t got a clue about preeclampsia, didn’t know what it was and then soon as I put my symptoms up and it’s in your face, you can’t ignore it. It’s like wow, it’s there, and you’re just like warning signs go off. But like if I hadn’t had that information I wouldn’t probably have been as worried, I wouldn’t have known what it was. Or have to know to go to the doctors. So it’s good and bad I suppose.

Participant 3, primiparous

### Pregnancy-Specific Focus

#### Online Information Seeking Different Now Pregnant

Some participants had indeed found that their online health seeking behavior had changed, in terms of searching more, since becoming pregnant. Perhaps, indicating that searching online for health and pregnancy information does indeed increase, or at least change, during pregnancy.

#### Baby Development

Many women enjoyed the website and mobile apps that informed them of their babies’ development and symptoms associated with pregnancy development.

### Reliable Sources

The participants in this study had specific sites and apps they viewed as reliable or official sources in which to seek pregnancy-related information from. The most popular website for pregnancy-related information was the National Health Service (NHS), with 2 women mentioning the NHS Start4Life in particular. Table 3 lists the sites the women used and felt where reliable sources of online information. Only 1 participant mentioned being recommended a site (Association for Improvements in the Maternity Services [AIMS]) by a midwife. Unsurprisingly the official NHS website was mentioned most reliable as it is the most widely known and reflects the current National Institute for Health and Care Excellence (NICE) guidance on treatment.

**Table 3:** Websites and apps used by the participants.

Website or app	n
NHS^a^ (NHS Start4Life)	16 (2)
The bounty app	8
Net mums	7
Mums net	4
Baby centre	4
Emma’s diary	1
AIMS^b^	1

^a^NHS: National Health Service.

^b^AIMS: Association for Improvements in the Maternity Services.

### Knowing When to Stop

In regard to any insights into how women skillfully surf, it was vital to gain an understanding and some insight into how pregnant women knew when to stop searching online for pregnancy-related health information. The most popular themes were (1) the information is no longer helpful or helping them, (2) saturation, and when the participant felt they were reading the same information and searches were no longer producing new information, but the same information was being repeated and (3)reassuring, in that they feel they had sought and gained an appropriate answer and they felt reassured. Other participants in the study indicated that they often found it difficult knowing when to stop:

It depends how worried I am about something. So I am a bit of a worrier like I do have anxiety so I will like sometimes go through a few pages and read everything. I’d probably carry on searching and sometimes make it worse. Because the more you search the further you go down the search results the more unclear and the more worrying the information gets.

Participant 14, multiparous

Whereas others found it much easier to get the information they required and could stop searching:

*I think sometimes it’s just about reassurance sometimes, reading it and thinking right okay, I’ll be satisfied with that. And then that will be done until something else.* [Participant 7, multiparous]

## Discussion

### Principal Findings

Knowing when to stop searching was different for different women. The Web was considered an extremely quick and accessible source, compared with offline official sources used for support and reassurance from those going through the same experience—normalizing and used for self-triage, possibly prior to seeking medical advice. The information sought online was not a replacement for a midwife or doctor’s advice, supporting previous findings [5,7]. In general, pregnant women tended to find the Web a useful source of information and advice during their pregnancy. Although there were things that worried them when they searched online, overall, the accessibility of online information and the stories and experiences of other women were viewed as reassuring. This reassurance was provided through both practical information and advice as well as through emotional support; both equally reassuring for the participants in this study.

The findings from this research indicate that searching online does indeed increase during pregnancy for women. However, this online searching was not necessarily a bad thing. It was clear that the women gained reassurance from hearing the stories and experiences of other women, either to gain information and knowledge or to gain a “second opinion.” This supports Bernhardt and Felter’s research on (female) parents’ health information seeking for their children [10]; allowing women to feel more in control and informed during their pregnancy [13]. This study does give an indication that women liked to have some form of control in their pregnancy. This is shown through women wanting to seek out information on their own, perhaps enabling them to feel empowered and informed with some level of control. This may be especially pertinent for women with complications or who have had complications in their pregnancy.

Not feeling as if they were the only ones experiencing something also normalized symptoms and issues for women, supporting previous research [7]. However, it was evident that this online information was not a substitute or a replacement for offline information [5,7], confirming the view [5] that information sought online is secondary in terms of both priority and reliability toward offline sources. However, in this study, women sought information online before going offline. In one particular instance, searching online convinced one woman that she did indeed need to seek offline care. In particular, women enjoyed the websites and mobile apps that informed them of their babies’ development and symptoms associated with pregnancy development, again supporting previous findings [7,10]. The Web, in particular the online forums for pregnant women, were viewed as good place to communicate and connect with others, providing women with a feeling of support as well as enabling women to develop networks with others going through pregnancy at the same time. This is valuable as women may not have this network of support offline.

However, the convenience of the Web and the fact that “Google it” is a well-known and established term of phrase, highlights that the Web is, for most people, a first port of call. This was found to be the case for pregnant women, who for one reason or other would turn to the Web first for answers or reassurance before seeking advice and reassurance offline. Numerous reasons were provided from the interviews as to why it was the first port of call aside from it being quick and accessible, namely, not wanting to bother their midwife or another HCP, wanting to be more informed about an issue or concern before going to see their midwife, not wanting to ask what may be deemed as silly questions when with their midwife, not wanting to waste their midwife’s time with a list of questions, not being able to contact or see a midwife and needing more speedy reassurance, feeling uncomfortable asking health professionals questions, or forgetting to ask certain questions when with the midwife. Online information provided short-term reassurance whereas information sought offline through a midwife or HCP provided long-term reassurance. Online information provided by a professional did also provide long-term reassurance but it is unclear from this research how the women knew if any claims of information coming from a HCP were indeed genuine.

Not surprisingly the NHS was the most reliable source of information for participants. Only one pregnant women in this study had been recommended another website by a HCP (in this case their midwife). It would be beneficial for health professionals to provide pregnant women with information on reliable online sources and highlight them to the potential dangers of forums. HCP should suggest suitable sites to empower people [12], especially since previous research highlighted this as a concern for midwives that more and more pregnant women are searching information online [13]. This and prior research [5] note the dangers of seeking health information online due to the element of bias toward having access to “worst case scenarios.” Interestingly, participants in the study did acknowledge that the worst case scenarios are an issue online and that this is an affordance of the Web, allowing access to a wider network of people who you would not have access to, and hear stories from, offline. This acknowledgement and insight suggests a good understanding of the Web and how to skillfully surf.

In some ways it was almost expected that when searching online about pregnancy-related issues, one would come across worst case scenarios or as some women referred to them, horror stories. The pregnant women in the study referred a lot to reading online forums, and it was particularly recognized that forums were a place people who have, or have had, problems tend to go to share their story. It was also recognized and accepted that healthy people do not generally go on forums to say all is well, unless it was in response to a worst case scenario in a supportive way, such as experiencing something similar and now having a healthy baby. This also leads to the acknowledgment of reliability of information, in that it was frequently acknowledged that anybody can post on forums and blogs about their issues and experiences and this is often unmoderated. However, these are people’s opinions and personal experience which may not apply to others and certainly not everyone. Again this shows a level of how skilful surfing is and how people navigate the vast amount of information online. Knowing what certain information online does and does not provide differs depending on the source of that information.

No real difference was evident from the findings of any difference in the online searching approaches or styles in terms of pregnancy status between primiparous and multiparous participants. However, the authors would suggest further research in this area to perhaps investigate any change in confidence or anxiety levels during pregnancy and how this may impact online searching. It was noted that 1 multiparous participant had noticed a difference in her online searching between her two pregnancies. As she knew much more during her second pregnancy that certain symptoms or concerns were normal, her previous experience had notably, to her, reduced her need to search for information online.

### Strengths and Limitations

This methodology did present a number of limitations. First, the sample was self-selecting and was only relevant to pregnant women who did use the Web for information and support. The sample may reflect only those who feel competent with Web-based activities and who have reasonable information technology (IT) literacy skills. Due to the inclusion criteria, the study missed out on the insights of pregnant women who did not consider Web-based resources as useful during pregnancy. The women in the study all talked about googling symptoms and reading posts from other women who had posted on forums. It would therefore be interesting for future work to understand the main issues posted on forums that pregnant women discuss and also how the information received via forums and other pregnant women’s stories in particular, impact on decision making during pregnancy. Previous research has considered the impact on decision making of online information [5]; however, more research is needed in this area especially in terms of support online.

A second limitation of the study is the sample size. Since this is a qualitative study, a sample size of 16 interviews is sufficient. However, triangulation of these findings with a larger more quantitative study would add weight to the findings discussed here. The authors have data from a Web-based questionnaire but this was beyond the scope of discussion in this paper but will add weight to these findings in the future. A major strength of this study is that not only did the authors conduct interviews with currently pregnant women but the health information and other demographic data was also gathered. Having knowledge of information pertaining to women’s pregnancy at the time of interview, such as trimester and medical conditions, enabled the researchers to consider factors which may influence use of the Web and how women searched. In terms of demographics, all but one of the women interviewed was white; perhaps a more ethnically diverse sample would yield differing results. In addition, this research gained knowledge of the participant’s experience of pregnancy-related complications, either during this pregnancy or a previous pregnancy. Previous research [6] has found health-related anxiety to increase in women during pregnancy who have experienced complications during pregnancy. More qualitative research into this area may provide insight into this area.

None of the women interviewed were in their first trimester of pregnancy. Due to the sampling technique employed, it was observed by the authors that the women approached to take part in the study who were in their first trimester of pregnancy did not want to be interviewed until they had reached the second trimester. This is understandable due to the increased risks of pregnancy loss in the first 12 weeks (first trimester). However, since the first trimester is a particularly worrying time for pregnant women and a time when symptoms such as morning sickness are often most persistent, it would be interesting for research to gain an insight of how women may change their online information or health searching during this period.

No longer helping, saturation, and feeling reassured where the main reasons why women stopped their online pregnancy-related searches. Women in the study felt confident they could navigate through the mass of data and there was an element of knowing when to stop searching. However, more research is needed to dig deeper and understand how pregnant women, and indeed other people seeking health information online, evaluate the quality of information sourced in order to gain a more complete insight into what constitutes skillful surfing.

### Conclusions

Searching for health information and advice online during pregnancy is viewed as quick, easy, and accessible. The affordances of the Web provide pregnant women the opportunity to go online as a first port of call. Knowing they were not alone, ascertained through the reading of online posts by other pregnant women sharing their experiences or symptoms, enabled women to normalize their experience and despite some caution required, was ultimately reassuring for pregnant women.

## Abbreviations

AIMS: Association for Improvements in the Maternity Services
HCP: health care professional
IT: information technology
NHS: National Health Service
NICE: National Institute for Health and Care Excellence
