# Correction: “You Sort of Go Down a Rabbit Hole...You’re Just Going to Keep on Searching”: A Qualitative Study of Searching Online for Pregnancy-Related Information During Pregnancy

**DOI:** 10.2196/jmir.8227

**Published:** 2017-07-13

**Authors:** Julie Prescott, Lynn Mackie

**Affiliations:** ^1^ Education and Psychology University of Bolton Bolton United Kingdom

The artice titled, “‘You Sort of Go Down a Rabbit Hole...You’re Just Going to Keep on Searching’: A Qualitative Study of Searching Online for Pregnancy-Related Information During Pregnancy” [J Med Internet Res 2017;19(6):e194], had several citation errors. These in-text citation errors have been corrected, as well as the resulting error in the numbering of the references, as follows:

“It would be beneficial for health professionals to provide pregnant women with information on reliable online sources and highlight them to the potential dangers of forums. HCP should suggest suitable sites to empower people [12], especially since previous research highlighted this as a concern for midwives that more and more pregnant women are searching information online [13].”

“This and prior research [5] note the dangers of seeking health information online due to the element of bias toward having access to “worst case scenarios.”

The old reference 13 (McManus) was deleted. The corrected and renumbered list of references appears below and in the corrected paper, re-published on the JMIR website on July 13, 2017, together with the publication of this correction notice. Because this was made after submission to PubMed, the correction notice has been submitted to PubMed. The corrected metadata have also been resubmitted to CrossRef.
